# Refractory Immune Thrombocytopenic Purpura with Abdominal Splenosis: A Complex Case

**DOI:** 10.1155/2023/9714457

**Published:** 2023-06-21

**Authors:** Joseph F. Mort, Danh T. Tran, Sean C. Dougherty, Robert Zielinski, Michael D. Williams, Kelly M. Davidson

**Affiliations:** ^1^University of Virginia, Department of Medicine, 1215 Lee Street Box 800466, Charlottesville, VA 22908, USA; ^2^University of Virginia, Department of Surgery, 1300 Jefferson Park Avenue, Charlottesville, VA 22903, USA; ^3^University of Virginia, Department of Medicine, Division of Hematology & Oncology, 1300 Jefferson Park Avenue Box 800716, Charlottesville, VA 22908, USA

## Abstract

Immune thrombocytopenia (ITP) is an acquired thrombocytopenia resulting from immune-mediated platelet destruction via antiplatelet antibodies and T cells. Medical management of ITP includes corticosteroids and multiple other adjunct therapies, with splenectomy generally being reserved for severe, refractory cases. In this clinical case report, we describe the evaluation of a 35-year-old male with a history of prior traumatic splenic injury who presented to the emergency department endorsing easy bruising and a petechial rash, ultimately found to have severe thrombocytopenia. The patient was diagnosed with primary ITP that proved to be refractory to a number of first- and second-line medical therapies. His course was complicated by the presence of abdominal splenosis discovered at the time of planned splenectomy and intra-abdominal hemorrhage requiring splenic artery embolization thereafter. To our knowledge, this is one of few published cases of ITP complicated by abdominal splenosis, highlighting the need to consider splenosis and the presence of accessory splenic tissue in cases of refractory ITP.

## 1. Introduction

Immune thrombocytopenia (ITP) is an acquired thrombocytopenia that occurs in children, young adults, and the elderly resulting from immune-mediated destruction of platelets [1]. Primary ITP, which accounts for approximately 80% of cases in adults, results from platelet destruction via antiplatelet antibodies and dysregulated T-cells [2–4]. Secondary ITP occurs in association with another underlying disorder, including human immunodeficiency virus infection, chronic hepatitis C infection, and autoimmune disease, among others [5]. The incidence of ITP is 3.3 per 100,000 adults per year. Women are more commonly affected in the young adult age range, but in the elderly, there is an even distribution among men and women. ITP is considered to be a diagnosis of exclusion; first-line management for ITP includes corticosteroids, most commonly pulse dexamethasone or oral prednisone with a taper. Second-line therapies with robust evidence supporting their use include thrombopoietin receptor agonists, rituximab, fostamatinib, and splenectomy [6].

Abdominal splenosis is an acquired condition in which small heterotopic implants of splenic tissue are autotransplanted within the abdomen [7]. Splenosis has been reported to occur in up to 67% of patients with a history of splenic trauma; however, its true incidence is unknown as it is often incidentally discovered at the time of surgery, on cross-sectional imaging, or at autopsy [8]. In addition to pathologic confirmation, Tc99m-labelled heat-denatured red blood cell (Tc-99m-DRBC) scans have also been used to identify implants as splenic tissue [9]. Although a benign condition, cases of abdominal pain, gastrointestinal bleeding, and recurrence of hematologic diseases following splenectomy have been attributed to splenosis [7, 10–12]. Specifically, a small number of cases of recurrent ITP in the setting of abdominal splenosis have been reported [13–15].

Here, we detail the presentation, evaluation, diagnosis, and management of a complex case of ITP refractory to multiple lines of medical management with attempted laparoscopic splenectomy complicated by the presence of abdominal splenosis.

## 2. Case Presentation

A 35-year-old man with a past medical history of traumatic splenic fracture (26 years prior to current presentation) presented to the emergency department with complaints of easy bruising in the bilateral lower extremities and two days of small volume epistaxis. A review of systems was negative for fever, chills, weight loss, night sweats, and other evidence of bleeding. Additional medical history included chronic headaches, for which he had been self-treating with ibuprofen daily; he was not taking any prescription medications or supplements. He denied alcohol, tobacco, and recreational drug use. Family history was notable for multiple first-degree relatives with ITP (father and sister); his father passed away from intracerebral hemorrhage related to ITP.

In regard to his history of traumatic splenic fracture, medical records indicated that at the age of nine, during a sibling altercation, he was thrown to the floor by his then 16-year-old brother and subsequently developed left-sided abdominal pain. He was brought to the emergency department with a physical examination notable for normal vital signs, a soft, nondistended, but slightly tender abdomen with pain on palpation of the left upper quadrant. A CBC was notable for a WBC count of 18.8 × 10^3^/*μ*L, hematocrit of 37.6%, and platelet count of 481 × 10^3^/*μ*L. A computed tomography (CT) scan of the abdomen and pelvis revealed a splenic fracture with a perisplenic hematoma and blood in the pelvis. He was managed with supportive measures, and procedural intervention was not required due to clinical stability. After a week of bed rest, he was discharged home. He did not have any further major interface with the healthcare system until the aforementioned presentation as an adult.

**Figure 1:**
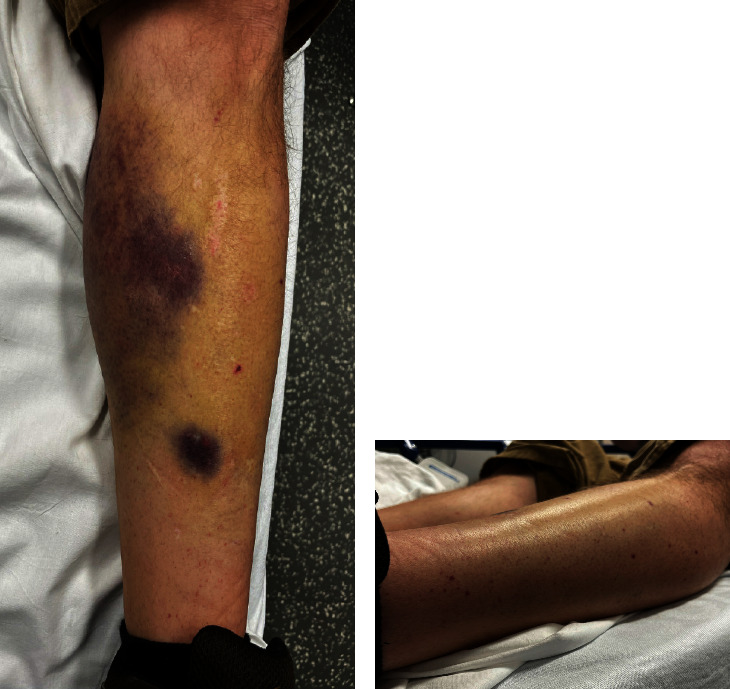
(a) Ecchymosis present on the medial side of the left lower extremity. (b) Petechial rash noted on the lateral portion of the left lower extremity.

On arrival, his vital signs were normal. Physical examination was notable for a large bruise on his medial left leg and petechiae on the bilateral lower extremities (Figures 1(a) and 1(b)) but was otherwise unremarkable. A complete blood count (CBC) revealed white blood cell count (WBC) of 6.14 × 10^3^/*μ*L, hemoglobin of 15.4 g/dL, and platelet count of 1 × 10^3^/*μ*L. After the transfusion of 2 units of platelets, the repeat platelet count was 4 × 10^3^/*μ*L. Peripheral blood smear revealed normal red blood cell morphology without schistocytes or Howell–Jolly bodies, severe thrombocytopenia, and the absence of giant platelets or platelet clumping. Initial laboratory work-up was normal (Table 1). Bone marrow biopsy was not performed due to high suspicion of ITP.

In the setting of severe isolated thrombocytopenia with minimal improvement despite multiple platelet transfusions, the nature of his clinical presentation, and an otherwise unremarkable evaluation, ITP was favored as the most likely diagnosis. The patient was started on dexamethasone 40 mg daily for four days and intravenous immunoglobulin (IVIG) 1 g/kg/day for two days. His platelet counts improved to 89 × 10^3^/*μ*L, and he was discharged. However, three days later, he developed an intractable headache and recurrence of epistaxis. His platelet count was noted to be 0 × 10^3^/*μ*L, and he was readmitted to the hospital. No intracranial bleeding was seen on the CT of the head. He received two additional days of IVIG at 1 g/kg/day and prednisone 80 mg daily with plans for a taper of 10 mg weekly. He also received avatrombopag 20 mg daily as a second-line agent, which was later increased to 20 mg daily alternating with 40 mg daily after discharge. He had transient improvement in his platelet count to 109 × 10^3^/*μ*L.h

**Figure 2:**
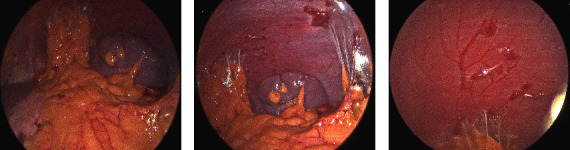
(a) Laparoscopic view of extensive omental adhesions present at the site of prior splenic injury. (b) Multiple sites of splenosis located around the spleen, left paracolic gutter, anterior peritoneum, and mesentery. (c) Multiple sites of revascularized ectopic splenosis.

**Figure 3:**
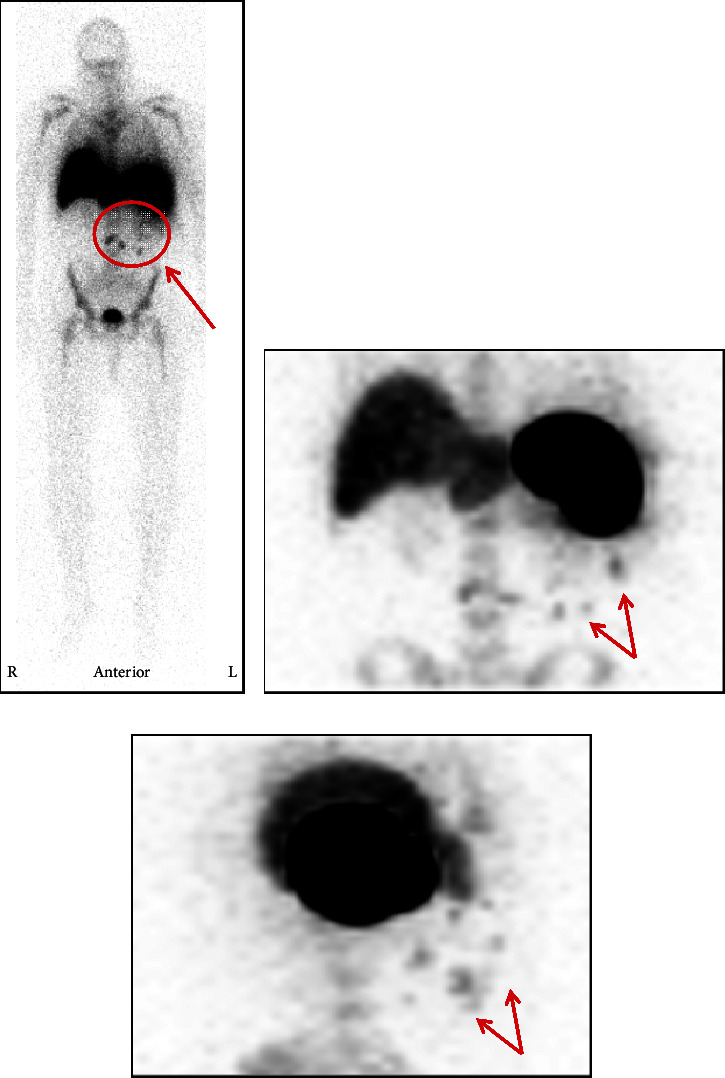
(a) Full-body tagged red blood cell scan approximately thirty minutes following injection of technetium 99 m red blood cells intravenously. Multiple foci at the splenic hilum (red circle). (b) Zoomed-in coronal view of sites of ectopic splenosis (red arrows). (c) Left sagittal view of multiple foci of splenosis posterior to spleen (red arrows).

Unfortunately, his platelet count decreased again to 6 × 10^3^/*μ*L when he was seen in the hematology clinic a few days after discharge despite the regimen above and two additional daily infusions of IVIG. During his third admission to the hospital, the patient received rituximab 375 mg/m^2^ weekly in addition to high-dose prednisone and avatrombopag. Due to the refractory nature of his thrombocytopenia, genetic work-up for inherited platelet disorders was initiated but was unrevealing. Despite the above interventions, his platelet count failed to improve, and it was recommended that he undergo a laparoscopic splenectomy; he was later taken to the operating room. Upon assessment of the spleen at the beginning of the case, several omental adhesions were found at the site of his prior splenic injury (Figure 2(a)). During adhesion take-down, extensive splenosis was noted around the spleen, left paracolic gutter, anterior peritoneum, and the mesentery (Figures 2(b) and 2(c)). The splenectomy was subsequently aborted as it was felt to be futile in the setting of extensive splenosis. Subsequent CT imaging with Tc-99m-DRBC corroborated the laparoscopic findings, with multiple intra-abdominal foci of splenic implants observed (Figure 3(a)–3(c)).

Given that splenectomy was delayed and might not be beneficial in the setting of splenosis, avatrombopag was switched to romiplostim 10 *μ*g/kg weekly, and mycophenolate mofetil 500 mg twice daily was initiated for further immunosuppression. However, his platelet count failed to improve and remained less than 10 × 10^3^/*μ*L over the next three days. His hospital course was then complicated by intra-abdominal bleeding from his recent surgical sites with a brisk downward trend of his hemoglobin to 7.8 g/dL from 14.0 g/dL four days prior. A CT angiogram of the abdomen and pelvis revealed high-density ascites concerning for intra-abdominal bleeding without active extravasation. He was started on an aminocaproic acid infusion and received three units of red blood cells and six units of platelets without any improvement in his blood counts and ongoing hemodynamic instability. A multidisciplinary decision was made to pursue interventional embolization of the splenic artery for auto-splenectomy to facilitate platelet recovery prior to open splenectomy, which was successfully performed on hospital day 14. After the procedure, he received two units of packed red blood cells and three units of platelets, and his platelet count improved to 267 × 10^3^/*μ*L within 48 hours.

**Figure 4:**
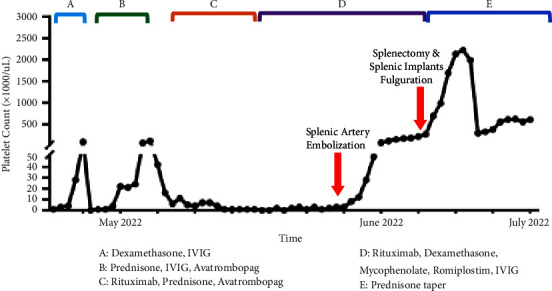
Platelet count trend throughout the patient's clinical course. Brackets correspond to therapeutic interventions for ITP as labeled below the chart.

The patient underwent open splenectomy on hospital day 16, during which peritoneal and mesenteric splenic implants were fulgurated with an argon beam coagulator. Postoperatively, the patient developed reactive thrombocytosis with a peak platelet count of 2,222 × 10^3^/*μ*L, which subsequently improved to a stable platelet count of 500–600 × 10^3^/*μ*L. Romiplostim and rituximab had been discontinued prior to open splenectomy, mycophenolate mofetil was discontinued two days prior to discharge, and prednisone was tapered two weeks after discharge. At a 2-month postdischarge follow-up in the hematology clinic, the patient was asymptomatic with a stable platelet count of 612 × 10^3^/*μ*L.  Figure 4 depicts his platelet count trend throughout his clinical course.

## 3. Discussion

Second-line therapies for corticosteroid-dependent or refractory primary ITP include thrombopoietin receptor agonists (TPO-RA), rituximab, fostamatinib, and splenectomy. These therapies should generally be initiated three months after diagnosis if the patient fails to respond to or remains dependent on corticosteroids [6, 16]. An individualized approach focusing on patient preference is recommended when deciding among these second-line options. Avatrombopag was selected as the second-line therapy for this patient given the rapid response rate of TPO-RAs, its oral administration, and its ability to be taken with food. The patient also had mild liver function test elevation at the time this agent was started contributing to its selection over eltrombopag, which causes higher rates of hepatotoxicity [17]. When possible, splenectomy should be delayed 12 to 24 months after diagnosis given the possibility of spontaneous remission within the first one to two years after disease onset [6, 16, 17]. Despite the increased risk of thrombosis and infection, splenectomy remains an effective intervention for corticosteroid-refractory ITP with initial response rates as high as 88% [6, 18]. Our patient's ITP proved to be highly refractory presenting with severe thrombocytopenia only days after initiation of corticosteroids and an initial response to IVIG. Despite early initiation of second-line medical therapies including avatrombopag, romiplostim, rituximab, and mycophenolate, he had no durable improvement in platelet count and splenectomy was considered before waiting the typical twelve months for spontaneous resolution given his critical condition and the need for rapid improvement in thrombocytopenia.

Approximately 20% of patients with corticosteroid-refractory ITP fail to respond to splenectomy, and 20–30% who had initial recovery of platelet count following splenectomy will relapse [6, 18]. Evaluation for an accessory spleen is recommended for patients with relapse of ITP postsplenectomy as it is relatively common (approximately 31–33% of these patients will have accessory spleens) [6, 19]. Accessory splenectomy has a good response rate for refractory ITP following splenectomy (50% in one study), and evaluation for accessory splenic tissue intraoperatively is recommended at the time of splenectomy [20, 21]. Because accessory spleens can occasionally be found in unusual locations such as the pancreatic tail, preoperative nuclear medicine scintigraphy with Tc-99m-DRBC can augment the identification and localization of accessory splenic tissue [22–24]. This imaging technique was utilized in our patient to identify ectopic splenic tissue prior to definitive surgical intervention. As opposed to an accessory spleen, however, our patient was found to have abdominal splenosis at the time of laparoscopic splenectomy complicating the management of his corticosteroid-refractory ITP.

Few cases of recurrent ITP in the setting of abdominal splenosis have been reported previously [13–15, 25, 26]. Mazur et al. present a case of a 25-year-old female with a history of splenectomy at age 12 for traumatic splenic rupture who underwent omentectomy and surgical removal of splenic implants when she developed steroid-refractory ITP. Preoperative 99mTc liver-spleen scan showed two areas of increased uptake in the left upper quadrant initially felt to represent accessory spleens, but at the time of laparotomy, splenosis with diffuse implants throughout the peritoneal cavity was seen. These implants were surgically resected. She had a resolution of thrombocytopenia postoperatively and was weaned from corticosteroids [13]. Toktas et al. present a case of a 40-year-old female with a relapse of ITP seven years following splenectomy for ITP and was noted to have a 6.8 × 3.1 mm intrahepatic mass on ultrasound and confirmed on computerized tomography. Initially felt to represent an accessory spleen, this was surgically resected with pathology more consistent with splenosis [14]. In addition to splenic rupture from abdominal trauma, splenosis can occur during laparoscopic splenectomy. This is exemplified in the case of a 12-year-old male who underwent laparoscopic splenectomy for refractory ITP with rupture of the retrieval bag leading to splenosis discovered 13 months later when he had a relapse of his ITP. He underwent omentectomy after splenic implants were found on laparoscopic exploration with the resolution of thrombocytopenia [25]. Another case of successful laparoscopic intervention for splenosis was reported by Barbaros et al. when a 32-year-old female who had previously undergone laparoscopic splenectomy for the treatment of ITP presented with a recurrence of ITP. Preoperative CT and Tc-99m-DRBC scans revealed multiple splenic tissue foci in the previously splenectomized region. At the time of surgery, splenic implants were seen on prior trocar sites, which were removed with the underlying fascia, and a section of the omentum containing several implants was resected. She had normalization of platelet count postoperatively and repeat scintigraphy showed no enhancing foci [15].

Another unique feature of our case is the use of splenic artery embolization as a temporizing measure for thrombocytopenia in the setting of postoperative intra-abdominal hemorrhage. Partial splenic embolization (PSE) is a procedural intervention that has been used to manage thrombocytopenia in patients with hypersplenism from portal hypertension in the setting of cirrhosis of the liver [27]. It has also been described as an alternative to splenectomy for second-line therapy of corticosteroid-refractory ITP [6, 28]. A retrospective analysis of 91 patients with steroid-resistant ITP who underwent PSE at a single center in Japan from 1988–2013 showed an overall response rate of 84% that persisted in 70% of patients at one year [28]. Although our patient's embolization procedure was not intended to provide durable remission of his ITP, it did result in a robust platelet response, which allowed him to proceed with open splenectomy and management of his splenosis.

Our case is unique in the identification of splenosis at the time of initial splenectomy for refractory ITP as opposed to the cases presented above where splenosis was identified years later in the setting of ITP relapse. After exploratory laparoscopy when the diagnosis of splenosis was made, the patient was able to undergo simultaneous splenectomy and management of splenosis with omentectomy and fulguration of splenic implants with argon beam coagulator. The technique of argon beam coagulation has been reported previously for the management of abdominal splenosis and was successful in this case as the patient had a durable improvement in thrombocytopenia postoperatively [29].

## 4. Conclusion

When ITP is refractory to second-line medical therapies, splenectomy should be considered. Evaluation for accessory spleens and splenosis should be performed to ensure the complete removal, as residual splenic tissue postsplenectomy is a common cause of ITP relapse. Scintigraphy with heat-damaged Tc99m-labelled red blood cells is a useful technique to identify accessory spleens or splenosis and specifically should be considered in patients with a history of splenic trauma. The use of partial splenic embolization for corticosteroid-refractory ITP requires further evaluation in prospective clinical trials but appears to provide a robust platelet response and may be an alternative to splenectomy in the future. Argon beam coagulation is another novel technique that appears to be effective for the management of widespread splenosis not easily amenable to surgical resection.

## Figures and Tables

**Table 1:** Laboratory workup of thrombocytopenia.

Test	Result	Reference range
Complete blood count		
White blood cells × 10^9^/L	6.14	4–11,000
Hemoglobin (g/dL)	14.5	12.0–16.0
Hematocrit (%)	43.0	35.0–47.0
Platelets × 10^3/^L	1	150–450
Complete metabolic panel		
Sodium (mmol/L)	141	136–145
Potassium	3.4	3.4–4.8
Chloride	105	98–107
Bicarbonate	23	23–31
Blood urea nitrogen (mg/dL)	13	10–20
Creatinine	1.0	0.6–1.1
Calcium	8.8	8.5–10.5
Alkaline phosphatase (U/L)	53	40–150
AST (U/L)	30	<35 U/L
ALT (U/L)	46	<55 U/L
Total bilirubin (mg/dL)	0.7	0.3–1.2
Miscellaneous		
Hepatitis B surface antigen	Negative	Negative
Hepatitis B core total antibody	Negative	Negative
Hepatitis C antibody	Negative	Negative
Hepatitis C viral load	Negative	Negative
HIV 1/2 antibodies and antigen	Negative	Negative
Vitamin B-12 (pg/mL)	434	210–815
Folate (ng/mL)	9.8	7.0–31.0
Lactate dehydrogenase (U/L)	221	125–250
Haptoglobin (mg/dL)	137	30–200
Reticulocyte count (%)	1.71	0.70–2.50
Reticulocyte count absolute	0.07	—
Immature reticulocyte fraction	10.6	1–100
Fibrinogen (mg/dL)	365	150–400
D-dimer (ng/mL)	<150	<243
Protime (sec)	11.9	9.0–13.0
Partial thromboplastin time (sec)	32.8	25.0–38.5
Thyroid-stimulating hormone	4.45	0.45–4.50
Antinuclear antibody	Negative	Negative
Helicobacter pylori	Negative	Negative
Parvovirus B19 Ab, IgG	Positive	Negative
Parvovirus B19 Ab, IgM	Negative	Negative
Epstein–Barr virus (EBV) capsid IgM	<0.2	<0.9
EBV capsid IgG	>8.0	<0.9
EBV heterophile IgM	<0.2	<0.9
EBV viral load	Negative	Negative
IgA (mg/dL)	249.0	63.0–484.0
IgG (mg/dL)	838.0	540.0–1,822.0
IgM (mg/dL)	357.0	22.0–240.0

## Data Availability

No data were used in the preparation of this case report.
